# Cultural differences in the beauty premium

**DOI:** 10.1038/s41598-025-02857-4

**Published:** 2025-05-21

**Authors:** Benjamin Kohler, Wladislaw Mill

**Affiliations:** 1 Center for Law & Economics, ETH Zurich, Haldeneggsteig 4, 8092 Zurich, Switzerland; 2 Department of Economics, University of Mannheim, L7 3-5, 68131 Mannheim, Germany

**Keywords:** Beauty premium, Language models, Cultural differences, Human behaviour, Evolution of language

## Abstract

A large body of research suggests that better-looking people are associated with various positive outcomes, an effect labeled the beauty premium. However, the vast majority of this evidence has been demonstrated in WEIRD (Western, Educated, Industrialized, Rich, and Democratic) countries, neglecting the role of cultural mechanisms in translating beauty into socio-economic outcomes. We close this gap by leveraging cultural differences embedded in an essential artifact of culture: language. Specifically, we establish a method for examining beauty associations in machine learning-based language models. Using this method, we replicate several standard findings in English. More importantly, we create a linguistic measure of the beauty premium and apply it to 68 languages. We provide new evidence that the beauty premium may be universal, with considerable heterogeneity across cultures. Furthermore, we discuss several new avenues for future research arising from our findings.

## Introduction

Physical appearance is a crucial characteristic with social and economic benefits to the mere attractiveness of individuals, widely known as the beauty premium^1–4^. While some research indicates that attractive people may face “beauty penalties,” and unattractive individuals may even receive benefits from “ugliness”^5^, most research suggest that more attractive individuals are generally evaluated and treated more positively^6^, are more likely to be hired^7^, persistently receive higher wages over their lifetimes^8^, receive better teaching quality ratings in academia^2^, and are even more likely to be elected in democratic elections^3^.

The explanations for the beauty premium are wide-ranging, from greater confidence affecting bargaining behavior^9^, stereotypical positive associations between beauty and positive personality traits^10^, and ascribed intellectual ability^11^, to evolutionary explanations stipulating that the beauty premium results from positive physical appearance signaling better genes and mate selection^12–14^. While most of these explanations suggest a universal beauty premium, research on the beauty premium has been conducted almost exclusively in WEIRD (Western, Educated, Industrialized, Rich, and Democratic) countries^15^, with only few studies focusing on the beauty premium in a non-WEIRD setting (i.e., China)^16,17^.

Although culture is highly predictive of social and economic behavior^18–21^, differences in the beauty premium across cultures have not been systematically examined. Culturally upheld values^22^, norms^23^, religious beliefs^24^, and narratives^25^ all serve as explanatory factors for the differences of market institutions and outcomes observed worldwide. Despite its potential relevance, the role of culture in social and economic effects of physical appearance has been largely overlooked in the literature. Even, cross-cultural comparisons examining *beauty stereotypes* are scarce and conducted on a small scale^26,27^. Moreover, these stereotypes have not been linked to the beauty premium, and more generally, there is no study to date investigating the beauty premium across cultures.

Our study fills this gap by examining how the beauty premium differs across cultures. To do so, we rely on a fundamental aspect of culture: language. Specifically, we use machine learning-based language models, called word embeddings^28^, trained on a vast amount of text data from all over the internet^29–31^, which enables us to 1) analyze the association between beauty and success in English and 2) investigate this association across a variety of cultures. Conceptually, word embeddings assign a vector to each word in a vocabulary, capturing the semantic relationships between words by positioning those that are more associated with each other (e.g., circle and ball) closer together in the multidimensional vector space than those that are less related (e.g., circle and cat). Thus, the geometrical similarity between two word vectors can be interpreted as their semantic similarity in the respective language. Word embeddings trained on a vast amount of diverse texts have been used to study specific settings (e.g. emotionality in US Congress’ speeches^32^, and economic training on judges ruling^33^), and cultural properties reflected in a language more broadly^34–37^. For example, it has been documented that implicit biases^34,38^ — particularly gender biases^36,37^ — social class associations^35^, and bodyweight-related stereotypes^39^ are all reflected within these word embeddings (see SI A for more detail). Following this literature, we identify the beauty premium in word embeddings. First, we extract relevant concepts based on the literature^34–36,39^, such as beauty, ugliness, and success. To capture the nuanced shades of meaning associated with our concepts of interest, we represent them by a set of words. Building on an existing framework^40^, we aggregate the strength of association between terms representing beauty to terms representing success and failure, effectively constructing a measure for the beauty premium within word embeddings. Finally, we apply our measure to word embeddings pre-trained on a large, diverse set of texts in 68 different languages to explore cross-cultural aspects of the beauty premium. This allows us to investigate the extent to which the beauty premium is ingrained in language usage worldwide.

There are two important findings from our analysis. First, we provide evidence that English word embeddings reveal a clear association between beauty and success. Thus, we establish that the beauty premium is indeed ingrained in the English language, which we call the *linguistic beauty premium*. On the other hand, we also find a stronger association of ugliness with failure, i.e., a linguistic ugliness penalty. Second, and most importantly, we focus on cultural differences in the beauty premium. The cross-language comparison reveals substantial heterogeneity in the existence and size of the linguistic beauty premium across cultures. The linguistic beauty premium ranges from non-existent in some languages (like Burmese and Vietnamese) to estimates up to four times larger than the English estimate (like Finnish and Japanese). However, the vast majority of languages reveal a clear and strong linguistic beauty premium. In summary, our findings suggest that a) the beauty premium is culturally embedded as reflected in language use, and b) this linguistic beauty premium varies across the world but is very persistently found around the globe.

## Results

### The linguistic beauty premium

**Figure 1:**
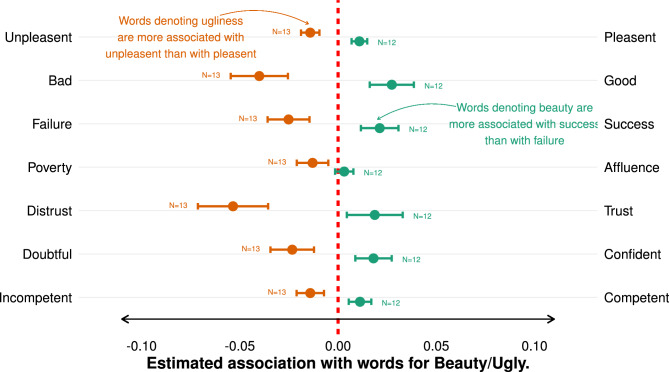
English *Beauty/Ugliness* association with other categories. The figure illustrates how *Beauty* and *Ugliness* are associated with a set of traits. Green dots denote the average association of all words representing *Beauty* on a two-poled scale (e.g., *Good* - *Bad* scale as a continuous measure). Red dots indicate the average association of all words representing *Ugliness* on the same scale. Positive values mean that words of either *Beauty* or *Ugliness* are, on average, more associated with the **right pol** of the scale (e.g., *Good*), while negative values mean that words of either *Beauty* or *Ugliness* are, on average, more associated with the **left pol** of the scale (e.g., *Bad*). Whiskers denote the 95% confidence interval.

First, to validate our approach, we test whether beauty is more associated with something 1) good and 2) pleasant^6^. To do this, we exploit the geometric similarity between word vectors as a measure of semantic proximity or association. We combine the average association of multiple, previously tested words representing *Beauty*/*Ugliness* (e.g., pretty, lovely, cute, etc.) with a target dimension (e.g., *Good* vs. *Bad*) using the POLAR framework (see Materials and Methods for more details on the POLAR framework and the word selection). The framework yields a single, interpretable association score for *Beauty*/*Ugliness* on the target dimension. In Fig. 1, we illustrate these association scores with several target dimensions (see SI B for the associations of the individual *Beauty*/*Ugliness*-words.). In this figure, green/red dots denote the average association of all the words representing *Beauty*/*Ugliness* on the respective dimension (e.g., *Good*/*Bad*). Positive values mean that *Beauty*- or *Ugliness*-words are, on average, more associated with the right pole of the dimension (e.g., *Good*), while negative values denote that *Beauty*- or *Ugliness*-words are, on average, more associated with the left pole of the dimension (e.g., *Bad*) (see SI C for regressions of the difference between *Beauty* and *Ugly* with regard to these dimensions). *Beauty*-words are, on average, much more associated with *Good*/*Pleasant* than with something *Bad*/*Unpleasant*. Similarly, *Ugliness*-words are, on average, much more associated with *Bad*/*Unpleasant* than with *Good*/*Pleasant*. The difference between *Beauty* and *Ugliness* on both dimensions is highly significant (*p* $\le$0.001). When focusing on all individual words (see SI B), we find that essentially all *Beauty*-words are closer, in terms of language space, to *Good* and *Pleasant* than to *Bad* and *Unpleasant*, with mirrored observations for all *Ugliness*-words. Thus, we find evidence that our construct of *Beauty*/*Ugliness* indeed reflects intuitive associations and is also consistent with the ’what-is-beautiful-is-good’ stereotype, whereby more attractive individuals are generally viewed more positively on a variety of traits^10,41^.

To obtain a measure of our main objective — the beauty premium — we estimate the association of *Beauty* on the *Success* - *Failure* dimension and, additionally, on the *Affluence* - *Poverty* dimension. *Beauty* is substantially more associated with *Success* and *Affluence* than with *Failure* and *Poverty*. *Ugliness*, on the other hand, is clearly associated with *Failure* and *Poverty*, revealing the presence of a linguistic ugliness penalty. These differences are again highly significant for both dimensions. Zooming in on the individual words representing *Beauty* and *Ugliness*, we find that the vast majority of the words resemble the same pattern. Therefore, we can conclude the existence of a beauty premium in the English language, highlighting that the association of beauty and success is deeply rooted in culture.

A major advantage of using language to study the beauty premium is that we can directly examine whether beauty is associated with common explanations for the beauty premium, specifically perceived competence^4,9,11^, trust^42–44^, and confidence^6,9^. For all three dimensions, we find the associations established in the literature. *Beauty* is more associated with *Competence*, *Trust*, and *Confidence* while *Ugliness* is associated with *Incompetence*, *Distrust*, and *Doubt*. Taken together, this evidence replicates — using the English language — some of the most common explanations for the beauty premium.

In SI D, we further validate that these results are specific to *Beauty*/*Ugliness*, by exploring two unrelated dimensions: *Fast*/*Slow* and *Far*/*Near*. We document that the *Fast*/*Slow* dimension does not exhibit the pronounced associations observed with *Beauty*/*Ugliness*, and that neither *Far*-words nor *Near*-words show meaningful associations with the relevant categories. These findings further support the conclusion that the linguistic beauty premium is specific to beauty. However, it remains possible that other words related to *Beauty*/*Ugliness* may exhibit similar patterns, as suggested by approaches in prior research see^45,46^.

### Cultural differences in the beauty premium

**Figure 2:**
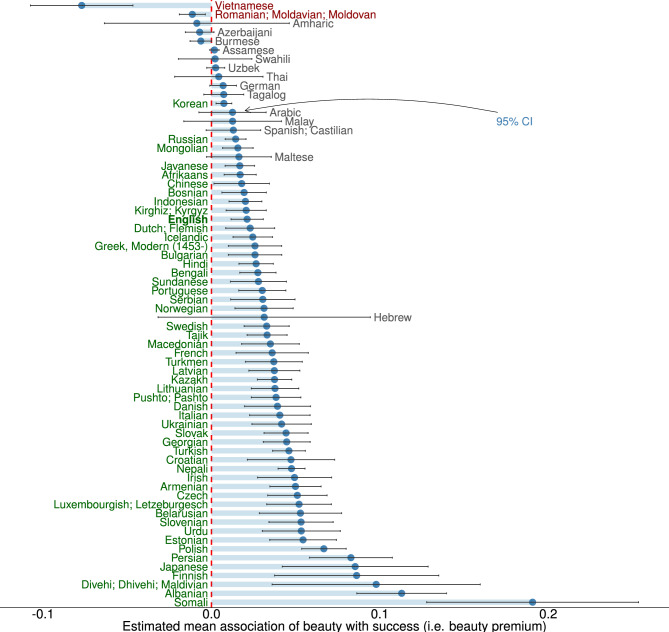
Linguistic beauty premium by language. The figure illustrates how *Beauty* is associated with *Success* relative to *Failure* (i.e., the linguistic beauty premium) across the 68 languages. Languages displayed in green denote a significantly positive linguistic beauty premium in the respective language. Languages displayed in gray and red denote a non-significant and a significantly negative linguistic beauty premium in the relative language, respectively. Whiskers denote the 95% confidence interval.

Next, we compare the linguistic beauty premium across cultures by comparing the association of *Beauty* between *Success* and *Failure* in each language word embedding. Figure 2 reports the linguistic beauty premium of all the 68 languages we use (see SI E for an estimation using a hierarchical linear mixed-effects model). The vast majority of languages (63 out of 68) show a positive and mostly significant association of beauty with success, suggesting that the beauty premium may be universal. Strikingly, we find substantial heterogeneity. For example, Hindi and French both have a stronger linguistic beauty premium than English. At the same time, some languages, like Burmese and Vietnamese, have even a negative association between *Beauty* and *Success*. From this, we can derive three main insights: (1) most languages show a linguistic beauty premium, (2) English is relatively mediocre in the extent of its beauty premium, and (3) some languages have no or even a negative linguistic beauty premium.

**Figure 3:**
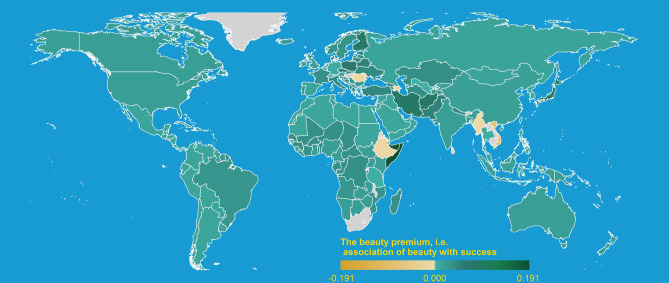
The linguistic beauty premium across the world. The primary language of each country is taken to establish the linguistic beauty premium. Green, ochar shaded countries indicate a positive and negative linguistic beauty premium, respectively. Grey-shaded countries speak, as their primary language, a language not part of our sample. Figure created with *R version 4.2.2*.

To study how the beauty premium is distributed across the world, we link the 68 languages in our sample to all countries using them as their primary language, illustrated in Fig. 3. With the exception of very few places (like Vietnam and Romania), we find a positive association between *Beauty* and *Success* across the world. Essentially, every continent makes this association (in SI F, we further zoom into Europe as we observe the biggest variation there). This result suggests an almost universal association of *Beauty* and *Success*, revealing a global beauty premium.

## Discussion and conclusion

In this study, we ask whether the beauty premium is reflected in language use and to what extent this culturally encoded beauty premium persists across the globe. Based on the POLAR framework^40^, we derive a meaningful representation of beauty and ugliness in word embeddings trained on large text corpora from various sources, allowing conclusions about general language use^34,35,47,48^. We demonstrate that this measure reflects basic stereotypes such as the association of beauty and pleasantness^6^. More importantly, we uncover a linguistic beauty premium and ugliness penalty in English word embeddings. We also show that biased beliefs about beauty, specifically greater trustworthiness, competence, and self-confidence, are also reflected in word embeddings. Finally, we find that the linguistic beauty premium is present in most of the languages studied but that the magnitude of the linguistic beauty premium shows considerable variance across languages.

By showing that the beauty premium is embedded in broader language use in many societies around the world, we highlight the importance of culture as a little-considered mediating channel for the beauty premium. Understanding the beauty premium as a culturally learned bias implies that the beauty premium may change as cultural norms, values, and narratives change, and attempts to mitigate the beauty premium must consider idiosyncratic cultural associations.

Our results provide strong support for — and a new perspective on — previously found biased beliefs as explanations for the beauty premium. The fact that these biased beliefs are reflected in language may explain why the beauty premium vanishes in experimental labor market settings when employers are informed about actual work performance^4^, but the beauty premium is still found in many real-world markets. While employers may temporarily update their beliefs, the cultural bias could serve as a default to which employers return when either the performance signals are ambiguous or when they face a long time between these signals and the bargaining situation^49^.

Our study joins recent work linking language use to economic outcomes^50,51^. First, by showing that the beauty premium is encoded in language use, our study highlights the importance of culture for the beauty premium. As language is not only a part of culture in itself but can reflect various cultural aspects such as norms or values, our study of language provides a cross-sectional view of the cultural beauty associations. Consequently, the stability of the beauty premium depends on the stability of the underlying cultural stereotypes, and policies attempting to mitigate the effects of the beauty premium should take into account the particularities of each culture.

Second, our research supports theories explaining the beauty premium as a result of beauty signaling individual traits^9–11,44^. By showing that associations between beauty and various traits found in experimental settings are replicated in word embeddings, the results suggest that the beauty premium may be rooted in entrenched cultural stereotypes.

Third, our results present new hypotheses for the size of the beauty premium for countries around the world, for example, comparing the size of the beauty premium in Japan and Vietnam. In addition, word embeddings could be used to find more universal and culturally specific associations of beauty. Thus, our approach can serve as a fruitful hypothesis-generating tool to find novel explanations for the beauty premium.

Our study is not without limitations. First, our method depends on the selected categories and words. We address this issue by using a multi-word representation and – where possible – previously established word sets. Further, we present our results for individual words and multiple categories, reducing the risk of selected results. Nevertheless. it might be possible that using different sets of words might lead to slightly different results. Second, our analysis only offers a static view on language. As cultural norms are subject to constant (but slow) change, we cannot rule out that our analysis depicts only a very recent status. Future research could analyze the stability of the linguistic beauty premium over time by training a word embedding enriched with temporal information. Third, by approximating cultural properties with language use, we miss cultural differences within the same language area. This especially limits the interpretability of languages with a widespread use in different cultures (e.g., French) and the detection of regional patterns. Given the lack of pre-trained word embeddings at the regional level and the trade-off between granularity and scope of regional analysis, we deliberately focused on establishing the novel linguistic beauty premium and applying it to a variety of languages spoken around the world. This approach is analogous to other research establishing a linguistic gender bias across languages^37^. However, we also encourage follow-up research to focus on certain demographic sub-groups, regions, and temporal dynamics of the linguistic beauty premium. Fourth, we study the cultural beauty premium using one specific – but highly relevant – factor of a culture: the language. However, we do not know whether and how our results would translate to other markers of culture. Future research might want to extend our approach to either other markers of culture or conduct other cross-cultural investigations into the beauty premium. Fifth, the evidence we provide is only correlational, and as such, we do not know the directionality of the effect. It seems plausible that some cultures have a specific view of beauty and success, which is then embedded in the corresponding language. However, it is equally possible that the language endogenously developed, leading to differential perceptions of beauty and success. To answer the causal question seems close to impossible at a large scale, but future research might be able to find causal empirical strategies identifying the direction of the effect. Sixth, our study focuses solely on providing evidence of a linguistic beauty premium. However, we do not address whether the relative strength of this premium predicts country-level outcomes, such as wages. Future research could explore this by identifying suitable variables recorded across a sufficiently large number of countries to extend our approach and examine whether and how the linguistic beauty premium translates into socio-economic outcomes.

Next to the suggested extensions, our study opens up other new avenues for future research. Our linguistic approach enables the simultaneous investigation of the existence and possible explanations of cultural influence on the beauty premium directly in real-world cultural traces at a relatively low cost. While we focused on detecting the linguistic beauty premium and existing explanations, our approach can serve as a blueprint for future research investigating new, culture-specific factors explaining the beauty premium. Training new word embeddings on corpora generated by a particular subgroup could also be used to study different linguistic beauty premia within the same language. Finally, our cross-lingual comparison provides hypotheses for a large number of regions without any prior information about the local extent of the beauty premium. Our data is, therefore, an invitation to test this hypothesis or to use it for further analyses.

Overall, we do not consider our study the last word on the matter but rather an initial piece of evidence on how culture might influence the beauty premium. This provides a direction for further research to create a more comprehensive understanding of the beauty premium.

## Materials and methods

### Data: pre-trained word embeddings

Our analysis utilizes pre-trained word embeddings (in particular, fasttext^52^). Word embeddings are vector representations of words arranged such that the distance between any two vectors mirrors their semantic relationship. This arrangement considers the context of each word in the corpus, in line with the linguistics distribution hypothesis, implying words derive meaning from their context^29^. The model iteratively adjusts vector distances based on word co-occurrence probability. These word embeddings are derived from extensive machine learning training on vast, diverse texts to reflect general linguistic features rather than specific textual properties^30^. These models developed for more than 100 different languages^31^, can serve as inputs to models fine-tuned for specific tasks^47^, and have demonstrated human-like notions of semantic relationships^30^. The underlying pre-trained word embeddings we use were trained on a mixture of texts from Wikipedia and large sets of texts collected from the web in the respective language^31^. While the size and sources of the training data vary widely between languages (from around 1.9 billion words in Hindi to 650 billion words in English), it is unlikely to have a significant impact on our results as 1) even the smallest corpus is vast, 2) the methodology of text collection is consistent between languages, and 3) these embeddings have previously been shown to represent fundamental cultural differences, such as gender bias^37^. Following the argumentation of previous research on bias in word embeddings, pre-trained models benefits our study by boosting replicability and representativeness due to adherence to best practices during training^34,35,37^. Moreover, these models have been trained on a large amount of data, likely representing a wide variety of language use within a given culture.

### Concept selection

To use word embeddings to study our objects of investigation – i.e., the beauty premium – we first identify key concepts of interest that represent the beauty premium based on the literature. The beauty premium broadly refers to the phenomenon that more physically attractive individuals receive certain benefits. We, therefore, replicate the beauty premium in word embeddings as the relationship between the concepts of beauty (represented by the antonym concepts *Beauty*/*Ugliness*) and success (*Success*/*Failure*) and affluence (*Affluence*/*Poverty*), respectively. Furthermore, the concepts of trust (*Trust*/*Distrust*), self-confidence (*Confident*/*Doubtful*), and competence (*Competence*/*Incompetence*) are selected to analyze the relationship between beauty and personal characteristics. Further, the positive concepts (*Good*/*Bad* and *Pleasant*/*Unpleasant*) have been used.

### Word selection

As common practice, we use multiple related words to represent concepts^34,35^. This approach captures various facets of a concept’s meaning, reduces bias from single terms, and allows the construction of confidence intervals for word selection sensitivity. While word and concept selection relies on informed judgment, we enhance our method’s robustness by using tested vocabulary from linguistic and psychological studies (see SI G for an overview of the vocabulary and sources). In order to study the beauty premium across languages, we first translated the selected words for each selected concept from English to the respective languages using an online translation tool. We then used these words in the respective languages to obtain the corresponding pre-trained word embeddings in these languages. We excluded regional accents and languages for which translation was not possible, resulting in word embeddings for 68 different languages.

### POLAR framework

We build on the POLAR framework for capturing cultural associations in word embeddings^40,53^. The POLAR framework is designed to add an interpretable dimension to the otherwise only relatively interpretable elements of pre-trained word embeddings. To illustrate, consider the linguistic beauty premium. We are interested in the association between the concepts *Beauty* and *Success*. We cannot obtain a meaningful interpretation of this association by a measure of similarity between these two concepts alone, as similarity can only be interpreted relative to other similarities. Therefore, we need to obtain the value of the concept *Beauty* on a *Success-Failure* dimension, i.e., is *Beauty* relatively more associated to *Success* or to *Failure* on a continuous *Success-Failure* scale. The POLAR framework offers such a score simultaneously for multiple vectors and dimensions^40^.

As a first step, we select a set consisting of pairs of word vectors, each representing a pole on a dimension. For example, for the *Success-Failure* dimension, we consider $\{$ $(\overrightarrow{successful}, \overrightarrow{unsuccessful})$, $(\overrightarrow{victorious},\overrightarrow{failed})$, $(\overrightarrow{winning},\overrightarrow{losing})$, $...\}$.

By calculating the vector difference between the opposite word vector pairs, we obtain the direction vector for each antonym pair. These direction vectors can be stacked to form a direction matrix:

$$Dir^{Success/Failure} = \begin{Bmatrix} \overrightarrow{successful}-\overrightarrow{unsuccessful}\\ \overrightarrow{victorious}-\overrightarrow{failed} \\ \overrightarrow{winning}-\overrightarrow{losing} \\ ... \end{Bmatrix}$$

We can get the values of a set of words with respect to each of the direction vectors by projecting their corresponding vectors, e.g., $\{$ $\overrightarrow{beautiful}$, $\overrightarrow{attractive}$, $\overrightarrow{handsome}$, $...\}$, onto all of the dimensions. The result is a single value for each of the words of interest ($\{\overrightarrow{beautiful}$ , $\overrightarrow{attractive}$, $\overrightarrow{handsome},...\}$) for all dimensions ($\{\overrightarrow{successful}$ - $\overrightarrow{unsuccessful}$, $\overrightarrow{victorious}$ - $\overrightarrow{failed}$, $\overrightarrow{winning}$ - $\overrightarrow{losing}$, $...\}$). A positive value of, e.g., $\overrightarrow{beautiful}$ projected onto the dimension $\overrightarrow{winning}-\overrightarrow{losing}$ can be interpreted as a stronger association of *beautiful* with *winning* than with *losing* and vice versa^40^. Since, in our case, the different dimensions in the direction matrix and the vectors of interest all represent an overarching concept (i.e., *Success*, *Failure*, *Beauty*), we can simply aggregate these measures. By averaging the values over the different dimensions, we obtain a single score for each *Beauty*-word, representing its value on the *Success-Failure* dimension.

Finally, we average all these scores again to obtain a single score representing the value of the concept *Beauty* on the *Success-Failure* dimension, i.e., the linguistic beauty premium. By averaging over multiple word scores, we ensure that the vocabulary length for each concept does not bias the results. We obtain confidence intervals and the relevant statistics for *Beauty* on each of the dimensions by treating each *Beauty*-word as one independent observation. For each *Beauty*-word we can also obtain confidence intervals by mutating the word-antonym pair combination to construct the direction matrix. In order to obtain p-values, we use the average score of each word on the relevant dimension (e.g., the *Success-Failure* dimension) and compare, using t-tests, whether words representing *Beauty* differ, in this score, from words representing *Ugliness*.

### The linguistic beauty premium on the country level

We use the POLAR framework to measure the linguistic beauty premium by placing *Beauty* on the *Success-Failure* dimension for each of the 68 languages we use. Thereafter, we associate each country with their primary spoken language (obtained from GeoNames.org). Using this approach gives us the linguistic beauty premium for most countries of the world.

## Supplementary Information

Supplementary Information.

## Data Availability

The data we use are pre-trained word embeddings freely available at https://fasttext.cc/.
