# Supplementary material for: Cultural differences in the beauty premium
Source: Sci Rep. 2025 May 21;15:17632. doi: 10.1038/s41598-025-02857-4 (PMC12095813; doi:10.1038/s41598-025-02857-4)
Supplement: Supplementary file 1 — Supplementary Information. [file 41598_2025_2857_MOESM1_ESM.pdf]

Supplementary Information for

CULTURAL DIFFERENCES IN THE BEAUTY PREMIUM

BY

BENJAMIN KOHLER

WLADISLAW MILL

A. Some applications of word embeddings in social sciences

Manifold research uses word embeddings to understand the properties of a more or less narrowly defined set of texts and actors. For example, it has been used to train word embeddings to understand the evolution of emotionality in US Congress' speeches over time [32]. Similarly, it was used to investigate the impact of economic training for judges by comparing their use of language before and after the training using word embeddings [33].

While this research employs word embeddings as a tool for studying a group of interest, other research utilizes word embeddings to capture human biases more generally. Word embeddings "learn" semantic relationships between words solely from the structure of the underlying corpus. This means there is no "ground truth" for the relationship between words. As a result, they can also unintentionally reflect undesirable properties from the corpus. Prominent work, mimics the structure of the Implicit Association Test to determine the relative difference between two target categories' relation to two opposing attributes (e.g., male vs. female associated terms in relation to family vs. career terms) [34]. Replicating findings from psychology, it is shown that implicit biases are reflected in word embeddings and that the strength of these associations correlates with real-world characteristics such as the female job participation rate. This paradigm has been extended in multiple ways. For example, it has been used to add the temporal dimension to investigate the evolution of gender and ethnic stereotypes over a century [36] and to extend the geographical scope to analyze gender bias in 25 languages [37] showing that the distributional properties of word embedding capture real-world properties such as women's participation rate in STEM fields also across languages.

Building on the discovery of harmful biases in word embeddings, a growing body of research has emerged that uses these biases to investigate entrenched cultural properties. This line of research is based on the understanding that language models are derived from cultural artifacts, specifically human-produced text. By exploring the properties of an embedding trained on a representative language corpus, researchers can investigate the broader use of language within a culture. This approach offers valuable insights into cultural properties beyond limited text analyses, providing a more comprehensive understanding of the culture encoded in language use. For example, the associations of social class in word embeddings [35]. Specifically, this work derives social class dimensions by averaging the vectors of two opposing groups of words, e.g., the opposing groups – cultured, cultured, polite, ... and uncultured, uncultured, rude, ... – represent a cultivation dimension. Word vectors of interest (e.g., different music genres) are then projected onto these dimensions. This projection is interpreted as their position between the two poles of the dimension (e.g., how cultivated classical music is compared to pop music). Using multiple dimensions, it allows to map granular class associations of different social groups and objects,

replicating fundamental sociological results. Compared to previous approaches [34], this approach also has the advantage that it allows for the interpretation of the differences between individual terms in relation to the specific dimension of interest. A shared characteristic among all methods used to detect bias and cultural associations in word embeddings is their construction of a socially relevant dimension of interest. This dimension is derived from a predefined set of words and then associated with one or more categories represented by specific terms.

B. *Beauty*/*Ugly* in association with individual categories

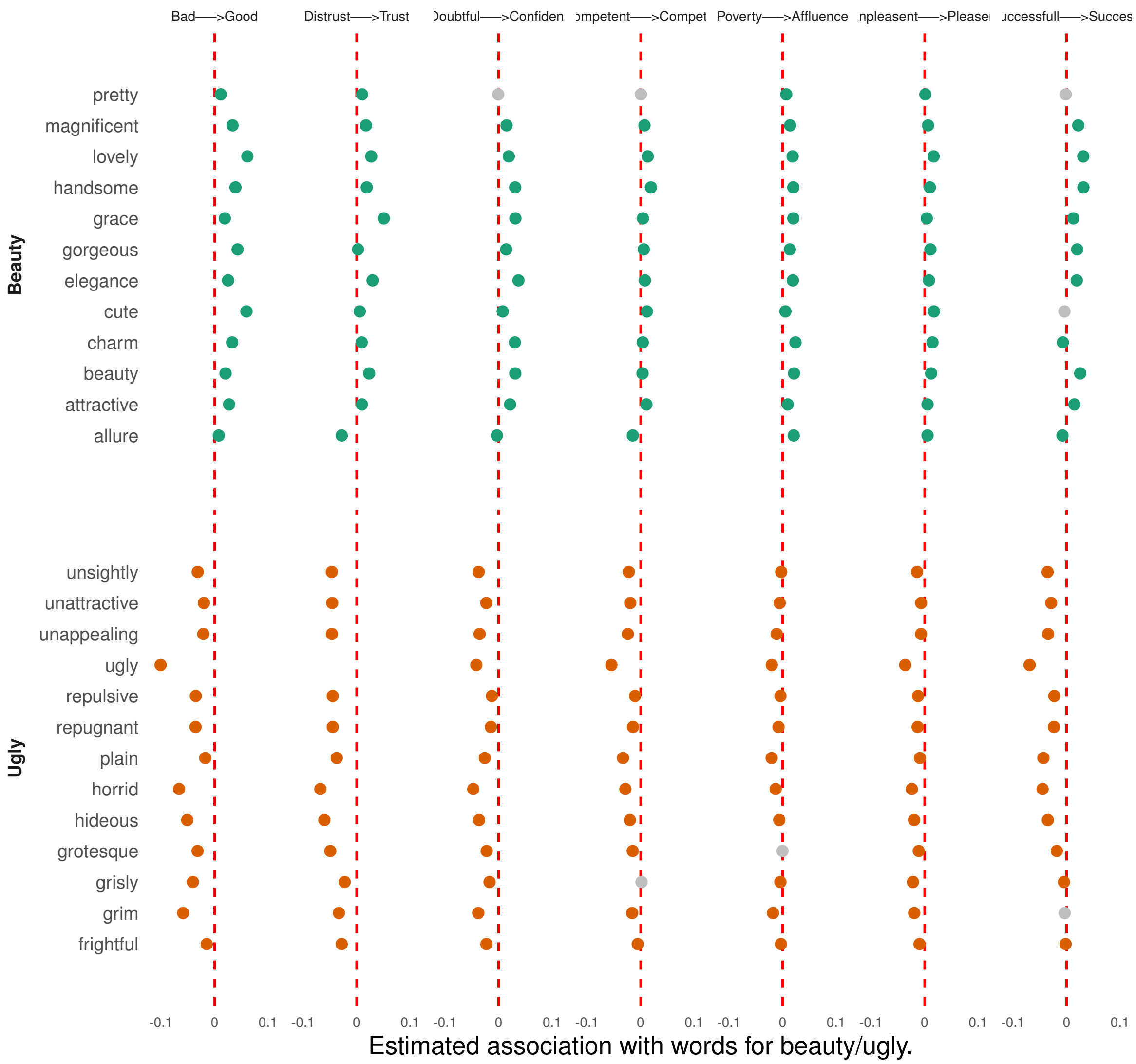

Figure S1: Individual English words of *Beauty*/*Ugly* in association with individual categories

The figure illustrates how individual words representing *Beauty* and *Ugly* are associated with individual categories. Green and red dots denote words representing beauty and ugly, respectively. Grey dots denote associations not significantly different from zero. Whiskers denote the 95% confidence interval.

C. Regressions on *Beauty*/*Ugly* associations with individual categories

	Association with:						
	Pleasant/Good		Success/Affluence		Competent/Confident/Trust		
	(1)	(2)	(3)	(4)	(5)	(6)	(7)
Beauty (Constant)	0.01***	0.03***	0.02***	0.003	0.01**	0.02**	0.02*
	(0.002)	(0.01)	(0.005)	(0.003)	(0.003)	(0.005)	(0.01)
Ugly	−0.03***	−0.07***	−0.05***	−0.02**	−0.03***	−0.04***	−0.07***
	(0.003)	(0.01)	(0.01)	(0.004)	(0.004)	(0.01)	(0.01)
Categories:	Pleasant⟷Unpleasant	Good⟷Bad	Success⟷Failure	Affluence⟷Poverty	Competent⟷Incompetent	Confident⟷Doubtful	Trust⟷Distrust
Observations	25	25	25	25	25	25	25
Adjusted R^2	0.76	0.72	0.67	0.35	0.60	0.61	0.66
Residual Std. Error (df = 23)	0.01	0.02	0.02	0.01	0.01	0.02	0.03
F Statistic (df = 1; 23)	78.15***	62.69***	49.54***	13.70**	36.89***	38.56***	46.62***
Note:							$^{+}p<0.10$; $^{*}p<0.05$; $^{**}p<0.01$; $^{***}p<0.001$;

Table S1: **Associations between *Beauty*/*Ugliness* and various categories in English.**

The table shows the estimates of linear regression of *Beauty* and *Ugly* relative to each of the dimensions. The observations denote the number of *Beauty* and *Ugliness* words (12 *Beauty* words and 13 *Ugliness* words.). Each column is an individual regression. For example, column 3 denotes the regression of how *Beauty* and *Ugliness* words are associated with success vs. failure.

D. Associations with unrelated categories

To assess the specificity of the linguistic beauty premium to the concepts of *Beauty*/*Ugliness*, we compare it to two unrelated dimensions: *Fast*/*Slow* and *Far*/*Near*. These concepts are largely independent of *Beauty*/*Ugliness*, making them suitable control groups. The analysis follows the same categorization as in Figure 1 in the main text.

Figure S2 presents the results for *Fast*/*Slow*. *Fast*-words are slightly more associated with something good, but not consistently so and often not significantly different from zero. Similarly, *Slow*-words show associations that are generally indistinguishable from zero. These findings indicate that the *Fast*/*Slow* dimension does not exhibit the pronounced associations observed with *Beauty*/*Ugliness*. Any positive effect for *Fast*-words is weak at best.

Figure S3 illustrates the results for *Far*/*Near*. Here, neither *Far*-words nor *Near*-words show meaningful associations with the relevant categories. The estimates are small and predominantly indistinguishable from zero, reinforcing the absence of a pattern similar to that observed for *Beauty*/*Ugliness*.

Together, these results demonstrate that the strong positive association of *Beauty* and the negative association of *Ugliness* are not mirrored in unrelated concepts. This suggests that the linguistic beauty premium is indeed specific to beauty.

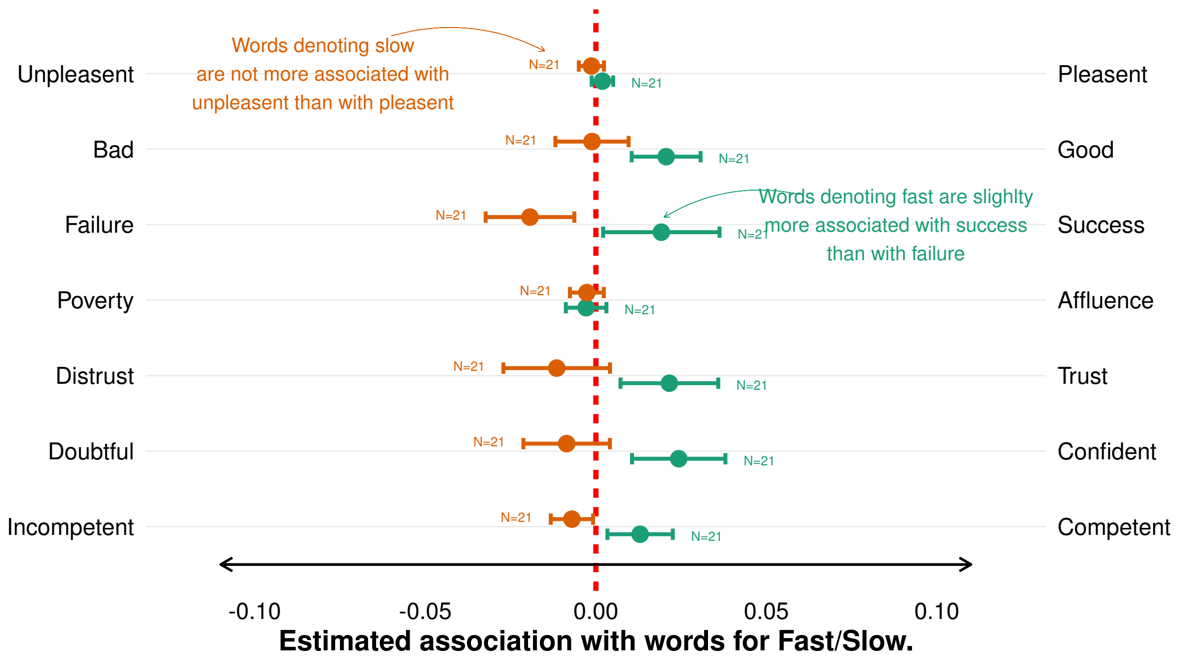

Figure S2: English *Fast/Slow* association with other categories.

The figure illustrates how *Fast* and *Slow* are associated with a set of traits. Green dots denote the average association of all words representing *Fast* on a two-poled scale (e.g., *Good* - *Bad* scale as a continuous measure). Red dots indicate the average association of all words representing *Slow* on the same scale. Positive values mean that words of either *Fast* or *Slow* are, on average, more associated with the **right pol** of the scale (e.g., *Good*), while negative values mean that words of either *Fast* or *Slow* are, on average, more associated with the **left pol** of the scale (e.g., *Bad*). Whiskers denote the 95% confidence interval.

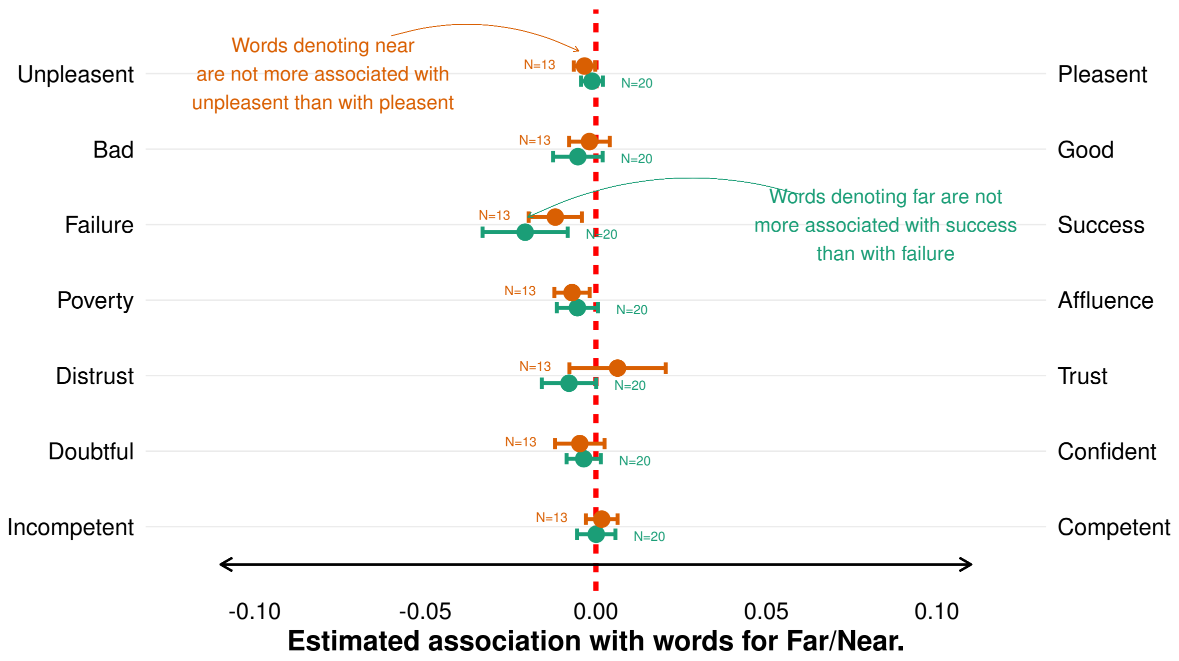

Figure S3: English *Far/Near* association with other categories.

The figure illustrates how *Far* and *Near* are associated with a set of traits. Green dots denote the average association of all words representing *Far* on a two-poled scale (e.g., *Good* - *Bad* scale as a continuous measure). Red dots indicate the average association of all words representing *Near* on the same scale. Positive values mean that words of either *Far* or *Near* are, on average, more associated with the **right pol** of the scale (e.g., *Good*), while negative values mean that words of either *Far* or *Near* are, on average, more associated with the **left pol** of the scale (e.g., *Bad*). Whiskers denote the 95% confidence interval.

E. Estimates of the linguistic beauty premium

	Linguistic beauty premium		
	Estimate	SE hierarchical	SE by language
Vietnamese	-0.077	(0.010)***	[0.014]***
Romanian; Moldavian; Moldovan	-0.011	(0.010)	[0.004]**
Amharic	-0.009	(0.010)	[0.025]
Azerbaijani	-0.007	(0.010)	[0.004]
Burmese	-0.006	(0.010)	[0.003]*
Assamese	0.002	(0.010)	[0.001]
Swahili	0.002	(0.010)	[0.010]
Uzbek	0.003	(0.010)	[0.002]
Thai	0.004	(0.010)	[0.012]
German	0.007	(0.010)	[0.004]
Tagalog	0.007	(0.010)	[0.005]
Korean	0.007	(0.010)	[0.002]***
Arabic	0.013	(0.010)	[0.009]
Malay	0.013	(0.010)	[0.013]
Spanish; Castilian	0.013	(0.010)	[0.007]
Russian	0.014	(0.010)	[0.003]***
Mongolian	0.016	(0.010)	[0.004]***
Maltese	0.016	(0.010)	[0.009]
Javanese	0.017	(0.010)	[0.004]***
Afrikaans	0.017	(0.010)	[0.004]***
Chinese	0.018	(0.010)	[0.007]*
Bosnian	0.019	(0.010)	[0.006]**
Indonesian	0.020	(0.010)	[0.004]***
Kirghiz; Kyrgyz	0.021	(0.010)*	[0.005]***
English	0.021	(0.010)*	[0.004]***
Dutch; Flemish	0.023	(0.010)*	[0.007]***
Icelandic	0.025	(0.010)*	[0.005]***
Greek, Modern (1453-)	0.026	(0.010)*	[0.007]***
Bulgarian	0.026	(0.010)*	[0.007]***
Hindi	0.027	(0.010)*	[0.005]***
Bengali	0.028	(0.010)**	[0.005]***
Sundanese	0.028	(0.010)**	[0.008]***
Portuguese	0.030	(0.010)**	[0.006]***
Serbian	0.031	(0.010)**	[0.009]***
Norwegian	0.031	(0.010)**	[0.008]***
Hebrew	0.031	(0.010)**	[0.029]
Swedish	0.033	(0.010)**	[0.006]***
Tajik	0.033	(0.010)**	[0.005]***
Macedonian	0.035	(0.010)***	[0.008]***
French	0.036	(0.010)***	[0.010]***
Turkmen	0.037	(0.010)***	[0.008]***
Latvian	0.037	(0.010)***	[0.007]***
Kazakh	0.037	(0.010)***	[0.005]***
Lithuanian	0.038	(0.010)***	[0.006]***
Pushto; Pashto	0.038	(0.010)***	[0.007]***
Danish	0.039	(0.010)***	[0.009]***
Italian	0.041	(0.010)***	[0.008]***
Ukrainian	0.042	(0.010)***	[0.008]***
Slovak	0.044	(0.010)***	[0.006]***
Georgian	0.045	(0.010)***	[0.006]***
Turkish	0.046	(0.010)***	[0.004]***
Croatian	0.047	(0.010)***	[0.012]***
Nepali	0.048	(0.010)***	[0.004]***
Irish	0.049	(0.010)***	[0.010]***
Armenian	0.050	(0.010)***	[0.007]***
Czech	0.051	(0.010)***	[0.008]***
Luxembourgish; Letzeburgesch	0.052	(0.010)***	[0.009]***
Belarusian	0.053	(0.010)***	[0.011]***
Slovenian	0.053	(0.010)***	[0.009]***
Urdu	0.053	(0.010)***	[0.011]***
Estonian	0.054	(0.010)***	[0.009]***
Polish	0.067	(0.010)***	[0.006]***
Persian	0.083	(0.010)***	[0.011]***
Japanese	0.085	(0.010)***	[0.020]***
Finnish	0.086	(0.010)***	[0.022]***
Divehi; Dhivehi; Maldivian	0.098	(0.010)***	[0.028]***
Albanian	0.113	(0.010)***	[0.012]***
Somali	0.191	(0.010)***	[0.029]***
Observations	816		
R^2	0.652		
F Statistic	20.640*** (df = 68; 748)		
Note:			*p<0.1; **p<0.05; ***p<0.01

Table S2: **Estimates of the linguistic beauty premium by language**

The table displays the estimated linguistic beauty premium by language (column one). The second column displays the standard error of the estimate using a hierarchical mixed-effects model accounting for word-specific random effects. The third column displays the standard error obtaing from simply aggregating the beauty premium by language. Stars indicate the corresponding levels of significance for the specific standard errors.

F. The linguistic beauty premium in Europe.

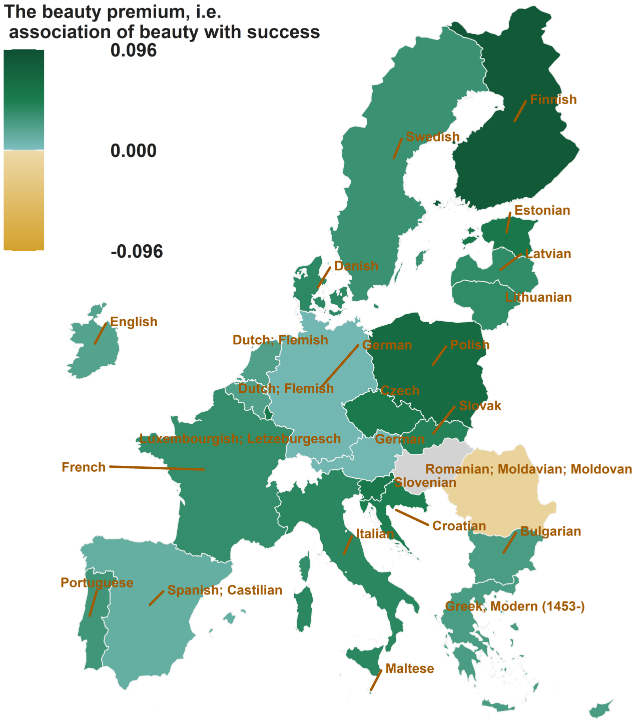

Figure S4: The linguistic beauty premium in Europe.

The primary language of each country is taken to establish the linguistic beauty premium. Green, ochar shaded countries indicate a positive and negative linguistic beauty premium, respectively. Grey-shaded countries speak, as their primary language, a language not part of our sample. Figure created with *R version 4.2.2*.

In the main part of the paper, we have seen that the linguistic beauty premium is present all over the world, with only very few countries showing a negative relationship. The continent with the seemingly biggest variation was Europe. Focusing on Europe has some advantages over other continents in terms of language use. First, Europe has a variety of different languages at close geographical proximity. Second, Europe is culturally very diverse, possibly allowing for substantial differences in the beauty premium. Third, the European countries have a high level of internet-penetration, and correspondingly, models of European languages have been trained on relatively large corpora. Therefore, in Figure S4, we illustrate the linguistic beauty premium across Europe. We clearly see substantial variation between countries. Eastern European languages (Polish, Slovak, Czech) have a strong positive association of beauty with success. On average, Eastern European languages seem to have a slightly higher association between *Beauty* and *Success* than non-Eastern European languages. We find that Eastern European languages do indeed have a higher association between *Beauty*-words and *Success* (β=0.01,t(406)=2.31, p= 0.022). This is even true if we account for *Beauty*-word-fixed-effects (β=0.01,t(408)=2.38,

p= 0.018). English, German, and Spanish also have a positive but moderate association between *Beauty* and *Success*. Interestingly, in Romania, the beauty premium seems to be reversed, as *Beauty* is (slightly) more associated with *Failure* than with *Success*. Overall, these patterns offer new patterns that might be testable in other empirical settings.

G. Words used for the categories

The following table illustrates all the words we used for each of the relevant categories. All the individual words and their translation into the 68 languages can be obtained from https://www.dropbox.com/scl/fi/oqnwhrslsdtmx9zbfq1h8/allvocabs.csv?rlkey=yb9d16lw2za1thiat1u6kl4l3&dl=0.

pleasant[1]	unpleasant[1]	good[2]	bad[2]	success[3]	failure[3]	poverty[3]	affluence[3]	competence[4]	incompetence[4]	self confidence[4]
caress	abuse	good	bad	successful	unsuccessful	poverty	affluence	competence	incompetence	assured
freedom	crash	love	disgust	victorious	failed	poor	rich	skillful	inept	bold
health	filth	glad	failure	triumphant	abortive	poorer	richer	capable	unskilled	assertive
love	murder	excellent	sadness	winning	losing	poorest	richest	proficient	ineffective	empowered
peace	sickness	magnificent	hurtful	thriving	failing	destitute	affluent	talented	inefficient	confident
cheer	accident	joyful	horrible	fruitful	fruitless	needy	advantaged	expertise	clumsy	self-esteem
friend	death	spectacular	dirty	prosperous	ineffectual	impoverished	wealthy	mastery	incapable	self-reliant
heaven	grief	cheerful	awful	success	failure	costly	economical	effective	unqualified	fearless
loyal	poison		abuse	win	lose	impecunious	exorbitant	knowledgeable	novice	secure
pleasure	stink					inexpensive	expensive	accomplished	ineptitude	self-assured
diamond	assault					ruined	exquisite	competent	incompetent	
gentle	disaster					necessitous	extravagant	efficient	untrained	
honest	hatred					skint	flush	qualified	bungling	
lucky	pollute					cheap	invaluable	proficiency	inadequate	
rainbow	tragedy					economical	lavish	skilled	unaccomplished	
diploma	bomb					penurious	luxuriant	adept	lacking	
gift	divorce					threadbare	luxury	adroit	amateurish	
honor	jail					unmonied	moneyed	able	fumbling	
miracle	poverty					indigent	opulent	resourceful	inexperienced	
sunrise	ugly					worthless	plush		untalented	
family	cancer					underprivileged	precious			
happy	evil					bankrupt	priceless			
laughter	kill					unprosperous	privileged			
paradise	rotten					underdeveloped	propertied			
vacation	vomit					insolvency	prosperous			

no self confidence[4]	trust[4]	distrust[4]	beauty[4]	ugly[4]	far[4]	near[4]	fast[4]	slow[4]
insecure	trust	distrust	beauty	ugly	far	near	fast	slow
doubtful	trusting	untrustworthy	allure	unattractive	distant	close	quick	gradual
uncertain	trustworthy	unreliable	charm	unappealing	remote	adjacent	rapid	deliberate
self-doubt	integrity	undependable	elegance	hideous	faraway	proximate	swift	sluggish
hesitant	reliable	fake	grace	repulsive	far-flung	nearby	speedy	leisurely
shy	dependable	unfaithful	lovely	frightful	removed	approaching	accelerated	unhurried
nervous	credible	disloyal	handsome	grim	outlying	neighboring	brisk	ponderous
self-conscious	faithful	deceptive	attractive	repugnant	beyond	imminent	fleet	languid
apprehensive			pretty	plain	isolated	bordering	nimble	sedate
unconfident			gorgeous	unsightly	aloof	intimate	hurried	plodding
			cute	grotesque	far-off	beside	agile	creeping
			magnificent	grisly	withdrawn	next-door	expeditious	tardy
				horrid	off	overhanging	immediate	cautious
					secluded		prompt	inactive
					inaccessible		snappy	torpid
					yonder		hasty	lagging
					detached		turbocharged	unrushed
					away		breakneck	slogging
					separate		lightning	snail-paced
					far-reaching		supersonic	glacial
							zooming	dragging

Note:
Sources of the Vocabulary
[1] Xu et al. (2014)
[2] Caliskan et al. (2016)
[3] Kozlowski et al. (2019)
[4] Authors
